# Contribution of adipocyte precursors in the phenotypic specificity of intra-articular adipose tissues in knee osteoarthritis patients

**DOI:** 10.1186/s13075-019-2058-9

**Published:** 2019-11-27

**Authors:** Florent Eymard, Audrey Pigenet, Cindy Rose, Anouchka Bories, Charles-Henri Flouzat-Lachaniette, Francis Berenbaum, Xavier Chevalier, Xavier Houard, Geoffroy Nourissat

**Affiliations:** 1 Sorbonne Université, INSERM, Centre de Recherche Saint-Antoine (CRSA), F-75012 Paris, France; 2 Department of Rheumatology, AP-HP Henri Mondor Hospital, F-94010 Créteil Cedex, France; 3 Department of Orthopedic Surgery, AP-HP Henri Mondor Hospital, F-94010 Créteil Cedex, France; 4 Department of Rheumatology, AP-HP Saint-Antoine Hospital, Labex Transimmunomics, DHU i2B, F-75012 Paris, France; 5 INSERM UMR-S 938 “Metabolism and Age-related Joint Diseases”, Saint-Antoine Research Center, 27 rue Chaligny, F-75571 Paris Cedex 12, France; 6 Groupe Ramsay Générale de Santé, Clinique Maussins Nollet, F-75019 Paris, France

**Keywords:** Osteoarthritis, Intra-articular adipose tissue, Preadipocyte, Inflammation

## Abstract

**Background:**

Intra-articular adipose tissues (IAATs) are involved in osteoarthritis (OA) pathophysiology. We hypothesize that mesenchymal cells residing in IAATs may account for the specific inflammatory and metabolic patterns in OA patients.

**Methods:**

Adipocyte precursors (preadipocytes and dedifferentiated fat cells (DFATc)) from IAATs (infrapatellar and suprapatellar fat pads) and autologous subcutaneous adipose tissues (SCATs) were isolated from knee OA patients. The ability of these precursors to differentiate into adipocytes was assessed by oil red O staining after 14 days of culture in adipogenic medium. The gene expression of adipocyte-related transcription factors (C/EBP-α and PPAR-γ) and development-related factors (EN1 and SFRP2) were analyzed. The inflammatory pattern was assessed by RT-qPCR and ELISA (interleukin 6 (IL-6), IL-8, Cox2, and prostaglandin E2 (PGE_2_)) after a 24-h stimulation by IL-1β (1 ng/mL) and by conditioned medium from OA synovium.

**Results:**

IAAT preadipocytes displayed a significantly higher ability to differentiate into adipocytes and expressed significantly more C/EBP-α mRNA than SCAT preadipocytes. IAAT preadipocytes expressed significantly less EN-1 and SFRP2 mRNA than SCAT preadipocytes. Unstimulated IAAT preadipocytes displayed a less inflammatory pattern (IL-6, IL-8, and Cox2/PGE_2_) than SCAT preadipocytes. In contrast, the response of IAAT preadipocytes to an inflammatory stimulus (IL-1β and conditioned media of OA synovium) was exacerbated compared to that of SCAT preadipocytes. Similar results were obtained with DFATc.

**Conclusion:**

IAAT adipocyte precursors from OA patients have a specific phenotype, which may account for the unique phenotype of OA IAATs. The exacerbated response of IAAT preadipocytes to inflammatory stimulation may contribute to OA pathophysiology.

## Background

Intra-articular adipose tissues (IAATs) have recently been identified as new actors in the pathophysiology of knee OA [1–5]. The strong association between obesity and knee OA [6] and the larger volume occupied by IAATs in the knee compared to other joints both suggested that these local adipose tissues (ATs) played a role in knee OA. Recently, we showed that the different IAATs from severe knee and hip OA share a common phenotype, which distinguishes them from autologous subcutaneous adipose tissues (SCATs). They are characterized by more fibrosis and more infiltration of inflammatory cells and, at the molecular level, by a less adipogenic and a more inflammatory pattern [1, 2, 5, 7]. Consequently, IAATs should be considered a specific subgroup of AT, such as subcutaneous, visceral, muscular, or perivascular AT [2].

Few studies have focused on the characteristics of IAAT resident cells to explain the specific phenotype of IAATs. In comparison with SCATs, there is an enrichment of the stromal vascular fraction (SVF) in the infrapatellar fat pad (IFP) of OA patients [5], which is associated with a slight increase in leukocyte infiltration [2]. Although macrophages are the main leukocytes found in IAATs, their proportion is similar in the SVF of the IFPs and SCATs [5], and the extent of macrophage infiltration in IAATs and SCATs varied in previous studies [2, 5, 8]. In contrast, mast cells are significantly increased whereas T cells are decreased in IAATs compared to SCATs [2, 5]. Thus, these contrasting data do not strongly support the unique involvement of the immune cell infiltrate in phenotypic differences between IAATs and SCATs. Moreover, IAAT adipocytes are smaller than autologous SCAT adipocytes [2], and IFP adipocytes release larger amounts of interleukin 6 (IL-6) and adipsin than SCAT adipocytes, suggesting that adipocytes are involved in the specific phenotype of IAATs [5].

Adipocytes are mesenchymal cells that originate from the differentiation of mesenchymal stem cells (MSCs) into adipocyte precursors (called preadipocytes) and then into mature adipocytes. To date, IAAT preadipocytes have never been characterized, and their role in OA has never been investigated. We hypothesized that the preadipocytes of IAATs from OA patients display unique features that may explain the adipocyte features and that may account for the unique phenotype of OA IAATs.

Intra-articular injections of MSCs from AT raise many hopes in the local treatment of OA. The first studies, in animals and humans, have shown that AT MSC injections or implantations had analgesic capacity and sometimes regenerative properties on cartilage [9–11]. Moreover, obtaining MSCs from AT is easier than from bone marrow. Although most basic and clinical studies were performed with MSCs from SCAT, some studies assessed the interest of MSCs from IFP. Thus, several authors showed that IFP MSCs displayed a higher chondrogenic potential compared to MSCs from other ATs [12–14] and had the ability to form neocartilage when cultured on a three-dimensional matrix [15]. First clinical studies also showed a positive symptomatic effect and a potential structural impact of IFP MSCs [16, 17]. The phenotypic characterization of adipocyte precursors from different IAATs of OA patients compared to autologous SCAT is therefore of interest because it could influence the choice of the best AT for obtaining MSCs for therapeutic purposes.

In the present study, we characterized the phenotype of adipocyte precursors from two different IAATs (IFP and suprapatellar fat pad, SPFP) and from autologous SCATs from knee OA patients. Both preadipocytes isolated from the SVF and dedifferentiated fat cells (DFATc) were studied. We compared their ability to differentiate into adipocytes and analyzed the expression of adipocyte-, development-, and inflammation-related factors according to the cell origin.

## Methods

### Adipose tissue samples

Tissues were harvested from patients with symptomatic end-stage knee OA undergoing total knee replacement at Henri Mondor Hospital (Créteil, France). Sequential patients from whom informed consent was obtained were included (*n* = 20). Patient characteristics are detailed in Additional file 3: Table S1.

After the incision of the superficial tissues and the opening of the articular capsule, the surgeon harvested the IFP and the SPFP. SCAT was harvested immediately below the incision. Tissues were stored in Dulbecco’s modified Eagle’s medium (DMEM) (Sigma Aldrich) with 1% bovine serum albumin (BSA) (Sigma Aldrich). This study was approved by the ethics committee of Henri Mondor Hospital and by the Assistance Publique Hôpitaux de Paris (approval no. 07-34) for biologic sample collection.

### Culture of preadipocytes derived from the stromal vascular fraction

ATs were carefully dissected as described previously [1]. For IAATs, special care was taken to separate ATs from the synovium. For each AT, vascular and fibrotic elements were removed. ATs were digested in 1 mg/mL collagenase (Roche Diagnostics) in DMEM with 4.5 g/L glucose, 100 U/mL penicillin (Sigma Aldrich), 0.1 mg/mL streptomycin (Sigma Aldrich), 15 mM HEPES (Sigma Aldrich), and 0.2% BSA for 1 h at 37 °C. AT digestion was then filtered through a 100-μm mesh filter and centrifuged for 6 min at 150*g*. After centrifugation, the SVF pellet containing preadipocytes sedimented at the bottom of the tube (Additional file 1: Figure S1) was resuspended after the removal of the supernatant and then harvested in 40 mL of red blood cell lysis buffer, consisting of 154 mM NH_4_Cl, 10 mM KHCO_3_, and 0.1 mM ethylene diamine tetraacetic acid (EDTA). After 10 min, another centrifugation (6 min, 150*g*) was performed, followed by the removal of the supernatant, which contained the lysed red blood cells. The pellet of preadipocytes was harvested in 40 mL of PBS containing calcium chloride (0.133 g/L) and magnesium chloride (0.1 g/L) (Sigma Aldrich). The solution was centrifuged again (6 min, 150*g*), and the pellet was harvested in DMEM (4.5 g/L) containing fetal calf serum (FCS) (10%) (Gibco), penicillin (100 U/mL), streptomycin (100 μg/mL), and 4 mM l-glutamine (Sigma Aldrich), and was placed in culture flasks (25 cm^2^) in an incubator (37 °C, CO2 = 5%). The media were changed every 48–72 h.

### Culture of dedifferentiated fat cells

The dissection, digestion, and filtration were performed as described above. After centrifugation (6 min, 1200 rpm), mature adipocytes, which contain the lipid droplet, did not sediment and floated on the top of the supernatant (Additional file 1: Figure S1). Adipocytes were collected, washed in PBS, and then recovered in DMEM (4.5 g/L) containing FCS (10%), penicillin (100 U/mL), streptomycin (100 μg/mL), and 4 mM l-glutamine. The cell suspension was then placed in a culture flask (25 cm^2^), which was completely filled to allow the floating adipocytes to contact the adhesive surface of the flask, as described by Matsumoto et al. [18] (Additional file 1: Figure S1). Thus, some adipocytes attached to the “ceiling” of the flask, initiating a dedifferentiation process and becoming DFATc. After 7 days, the medium was changed, and the flasks were inverted to allow DFATc proliferation to continue. DFATc culture was carried out at 37 °C in an incubator (37 °C, CO2 = 5%). The media were changed every 48–72 h.

### Flow cytometry

Preadipocytes and DFATc from passage 4 were seeded in a 10-cm petri dish in growth medium until confluence. Cells were then washed with PBS, and trypsinized and resuspended (5 × 10^6^ cells/mL) in staining buffer (PBS, sodium azide 0.02%, and serum bovine albumin 0.2%). Cells (100 μL) were incubated 20 min with propidium iodure and the following anti-human primary antibodies: CD31-PB, CD45-KO, CD90-APC, and CD105-PC7 (all from Beckman Coulter). After incubation, cells were washed twice with PBS, centrifuged 5 min at 150*g*, and resuspended in staining buffer (0.5 mL). Matrix of compensation was generated using VersaComp Antibodycapture Beads kit (Beckman Coulter), according to the manufacturer’s instructions. Data were acquired using Gallios (Beckman Coulter), and analysis was performed using Kaluza analysis software (Beckman Coulter).

### Stimulation of preadipocytes and dedifferentiated fat cells

Preadipocytes and DFATc from passage 4 were seeded in a 12-well plate (5 × 10^5^ cells/well) in growth media (DMEM 4.5 g/L + FCS 10% + penicillin (100 U/mL) and streptomycin (100 μg/mL) + 4 mM l-glutamine). After 48 h, cells were washed twice with PBS and were incubated for 24 h in 1 mL of similar medium without FCS with or without IL-1β (1 ng/mL) (Sigma Aldrich) or conditioned medium of the inflammatory part of the synovium from OA patients (150 μL conditioned media and 850 μL of culture medium without FCS), which was prepared as previously described [19]. After 24 h, conditioned media were collected and stored at − 80 °C. Cells were lysed by adding 148.5 μL of RLT Buffer (Qiagen) and 1.5 μL of β-mercaptoethanol to each well, RNA was extracted, and samples were frozen at − 80 °C.

### Adipocyte differentiation

Preadipocytes and DFATc were incubated at passage 4 in 12-well plates with adipogenic medium (50% DMEM 4.5 g/L, 50% F12 medium (Sigma Aldrich), penicillin (100 U/mL), streptomycin (100 μg/mL), 1 μM rosiglitazone (Sigma Aldrich), 50 nM insulin (Sigma Aldrich), 15 mM HEPES pH 7.4), and also IBMX 2.5 μM (Sigma Aldrich) and dexamethasone 100 nM (Sigma Aldrich) during the first 4 days. The media were changed every 48–72 h for 14 days.

### Quantification of adipocyte differentiation

After 14 days of culture, adipocyte precursors were washed twice with PBS and then incubated for 24 h in 1 mL of DMEM 4.5 g/L containing penicillin (100 U/mL), streptomycin (100 μg/mL), and 4 mM l-glutamine. After two washes, cells were fixed with 3.7% paraformaldehyde (PFA) (Sigma Aldrich). After 1 h, the PFA was removed, and two washes with 60% isopropanol (Sigma Aldrich) were performed. Two hundred and fifty microliters of diluted oil red O solution (stock solution of oil red O (1.7 mM in 200 mL of isopropanol) was diluted by adding 40% H2O and then filtering for 20 min before use) was added to each well for 10 min before washing three times with H_2_O. Finally, 1 mL of 100% isopropanol was added for 10 min to dissolve the fixed oil red O. This solution was collected, and the intensity of oil red O staining was spectrophotometrically measured at 450 nm, either immediately or after freezing at − 20 °C. One hundred percent isopropanol served as a negative control.

### Total RNA extraction and quantitative RT-PCR

Total RNA was isolated by using a Reliaprep RNA Cell miniprep system (Promega). RNA (250–1000 ng) was reverse transcribed by using an Omniscript RT kit (Qiagen). Gene expression was analyzed by quantitative RT-PCR with a Roche Diagnostics LightCycler 480 in a 12-μL final volume with specific primers (10 μM) (Additional file 3: Table S2) and GoTaq PCR Master Mix (Promega). PCR amplification included a denaturation step (5 min at 95 °C) followed by 40 cycles of 10 s at 95 °C, 15 s at 60 °C, and 10 s at 72 °C.

For each PCR, duplicates for each cDNA were run in parallel, and serial dilutions of a cDNA mixture were tested for each primer pair to generate a standard linear curve, which was used to estimate the amplification efficiency. The relative mRNA expression for all genes analyzed was normalized to that of 18S RNA (used as the internal reference gene) and was analyzed by using the efficiency method with Light Cycler 480 software.

### Enzyme-linked immunosorbent assay

ELISA kits were used to determine the concentrations of IL-6, IL-8 (both from Sanquin-PeliKine), and prostaglandin E2 (PGE_2_; Cayman Chemical) in conditioned medium.

### Statistical analysis

The non-parametric Wilcoxon signed-rank test was used to compare paired observations. To analyze the cell response to conditioned medium of OA synovium, non-parametric Friedman and Dunn’s multiple comparison tests and non-parametric non-paired Mann-Whitney test were used. Analyses were performed with GraphPad Prism 5 (GraphPad Software Inc., San Diego, CA, USA). Statistical significance was set at *p* < 0*.*05.

## Results

### More adipocyte differentiation and less basal expression of inflammatory factors were observed in IAAT preadipocytes than in SCAT preadipocytes

**Fig. 1:**
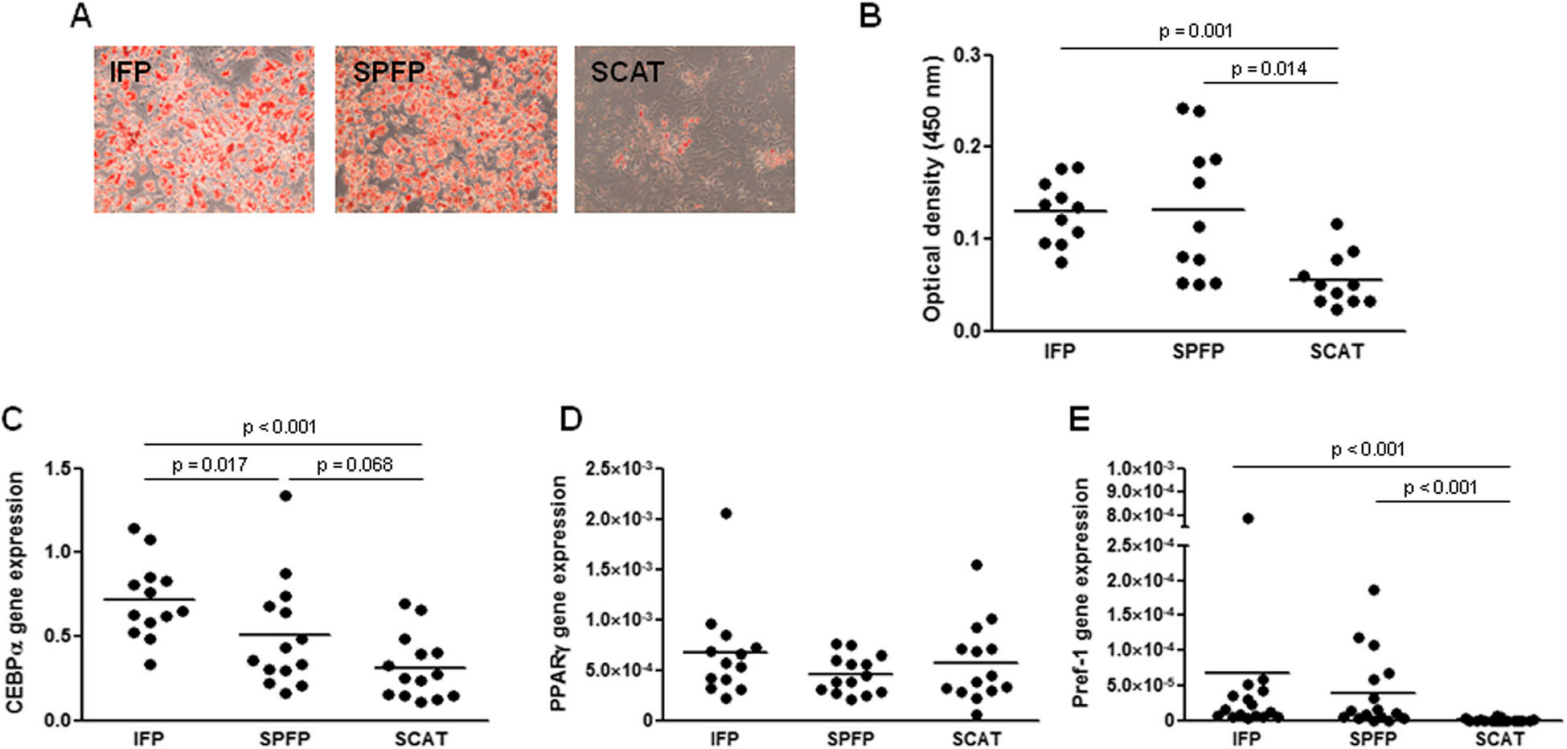
Adipocyte differentiation of preadipocytes isolated from IAAT and SCAT. **a**, **b** Preadipocytes from IFPs, SPFPs, and SCATs of OA patients (*n* = 11) were cultured for 14 days in adipogenic medium before the evaluation of adipocyte differentiation by oil red O staining (**a**) and staining quantification (**b**). **c**–**e** Relative mRNA expression of C/EBPα (**c**), PPARγ (**d**), and Pref-1 (**e**) by IFP and SPFP preadipocytes compared to the corresponding SCAT preadipocytes from OA patients (*n* = 12–15). The relative mRNA expression was normalized to that of 18S RNA. Bars indicate the mean expression levels

Preadipocytes were isolated from the SVF of IFPs, SPFPs, and SCAT of 17 patients. Flow cytometry experiments showed that preadipocytes from IFPs, SPFPs, and SCAT expressed the CD90 and CD105 mesenchymal markers and were negative for the leukocyte and endothelial markers CD45 and CD31 (Additional file 2: Figure S2). Most cells differentiated into adipocytes when cultured into adipogenic medium for 14 days (Fig. 1a, b). However, both IFP- and SPFP-derived cultures accumulated more lipid droplets than SCAT preadipocytes during adipocyte differentiation (2.9-fold, *p* = 0.001, and 2.7-fold, *p* = 0.014, for IFP- and SPFP-derived cells, respectively) (Fig. 1a, b). Consistently, IFP and SPFP preadipocytes expressed higher amounts of C/EBPα mRNA than SCAT preadipocytes (2.3-fold, *p* < 0.001, and 1.6-fold, *p* = 0.068, for IFP and SPFP, respectively) (Fig. 1c). In contrast, no significant difference in the expression of PPARγ was observed in the different cell types (Fig. 1d). The expression of the early marker of adipocyte differentiation Pref-1 was significantly higher in preadipocytes from IFPs and SPFPs than from SCATs (*p* < 0.001) (Fig. 1e). Pref-1 expression was observed in IFP preadipocytes from all patients and in SPFP preadipocytes from 14/16 patients, whereas it was expressed in SCAT preadipocytes from only 4/16 patients.

**Fig. 2:**
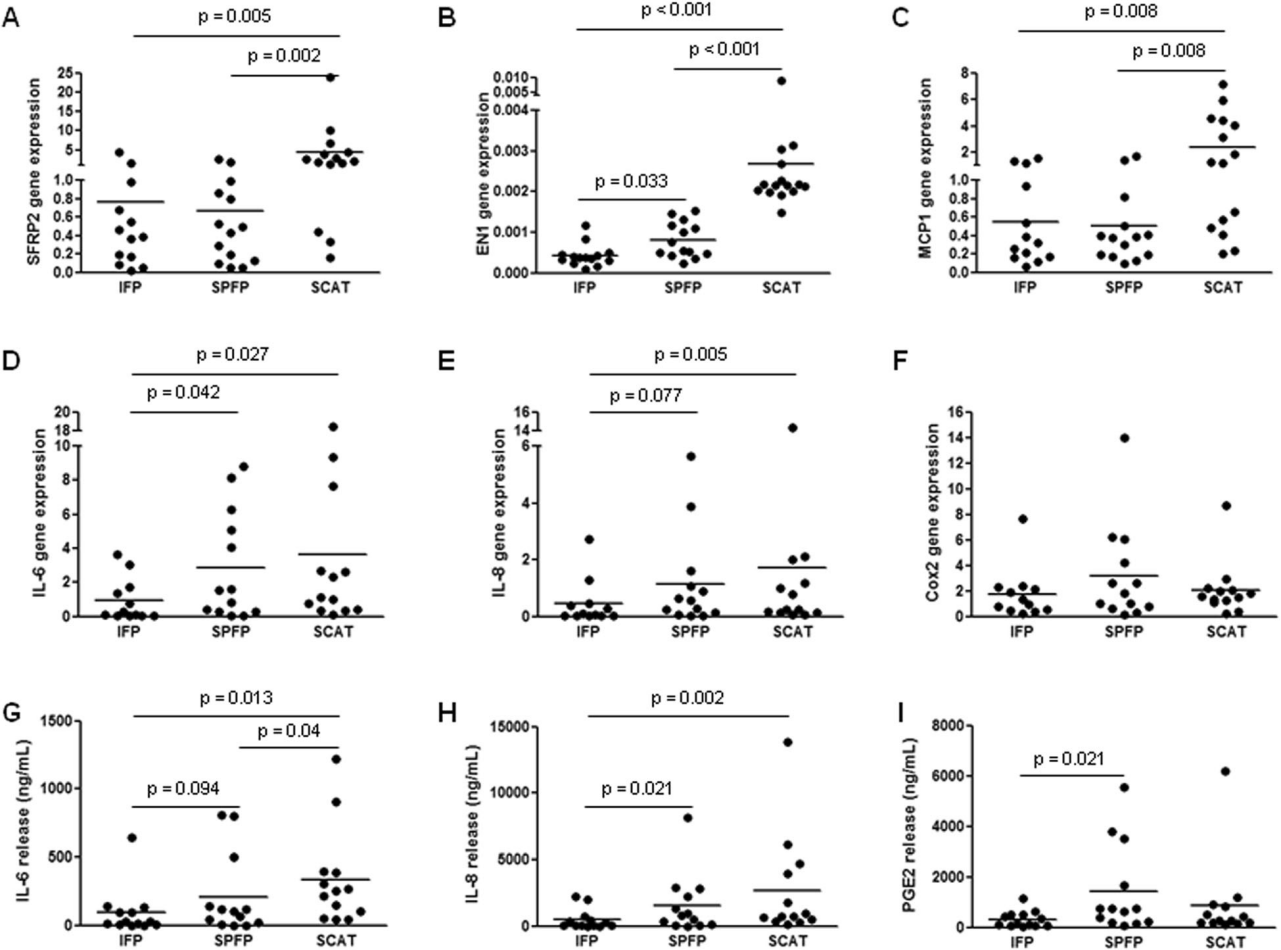
Molecular characterization of preadipocytes isolated from IAAT and SCAT. **a**–**f** Relative mRNA expression of SFRP2 (**a**), EN-1 (**b**), MCP1 (**c**), IL-6 (**d**), IL-8 (**e**), and Cox2 (**f**) in IFP and SPFP preadipocytes compared to the corresponding SCAT preadipocytes from OA patients (*n* = 12–15). The relative mRNA expression was normalized to that of 18S RNA. **g**–**i** The release of IL-6 (**g**), IL-8 (**h**), and PGE_2_ (**i**) by preadipocytes isolated from IFPs, SPFPs, and SCATs (*n* = 13). Bars indicate the mean expression levels

In addition to genes associated with adipocyte differentiation, the development-related genes EN1 and SFRP2 were also differentially expressed between preadipocytes from IAATs and SCATs, with a significantly lower expression of EN1 (0.2-fold, *p* < 0.001, and 0.3-fold, *p* < 0.001, for IFP and SPFP, respectively) and of SFRP2 (0.2-fold, *p* = 0.005, and 0.1-fold, *p* = 0.002, for IFP and SPFP, respectively) in IAAT preadipocytes than in SCAT preadipocytes (Fig. 2a, b). Since we and others have previously showed that OA IAATs produce more inflammatory factors than SCATs [1, 2, 5], we next compared the inflammatory phenotype of preadipocytes (Fig. 2c–i). The basal expression of MCP1, IL-6, and IL-8 by IFP preadipocytes was significantly lower than that of SCAT preadipocytes (*p* = 0.008, *p* = 0.027, and *p* = 0.005 for MCP1, IL-6, and IL-8, respectively) (Fig. 2c–e). IFP preadipocytes also expressed less IL-6 and IL-8 mRNAs than SPFP preadipocytes (*p* = 0.042 and *p* = 0.077 for IL-6 and IL-8, respectively) (Fig. 2d, e). SPFP preadipocytes expressed significantly less MCP1 mRNA than SCAT preadipocytes (*p* = 0.008), whereas no difference in the basal mRNA expression of IL-6 and IL-8 by SPFP and SCAT preadipocytes was observed (Fig. 2c–f). In accordance with mRNA expression, IFP preadipocytes secreted the lowest levels of inflammatory factors (Fig. 2g–i). SCAT preadipocytes released significantly more IL-6 and IL-8 than IFP preadipocytes (3.6-fold, *p* = 0.013, and 5.5-fold, *p* = 0.002, for IL-6 and IL-8, respectively) (Fig. 2g, h). In contrast, the levels of secreted inflammatory factors by SPFP preadipocytes were intermediate between those by IFP and by SCAT preadipocytes. SPFP preadipocytes released more IL-6, IL-8, and PGE_2_ than IFP preadipocytes (2.3-fold, *p* = 0.094; 3.2-fold, *p* = 0.022; and 4.4-fold, *p* = 0.022 for IL-6, IL-8, and PGE_2_, respectively) but significantly less IL-6 than SCAT preadipocytes (0.6-fold, *p* = 0.040) (Fig. 2g–i).

Together, these results indicate that the secretion pattern of inflammatory mediators and the differentiating factors expressed by preadipocytes from OA IAATs and SCATs are not similar, suggesting different phenotypes.

### Dedifferentiated fat cells display a phenotype similar to preadipocytes

**Fig. 3:**
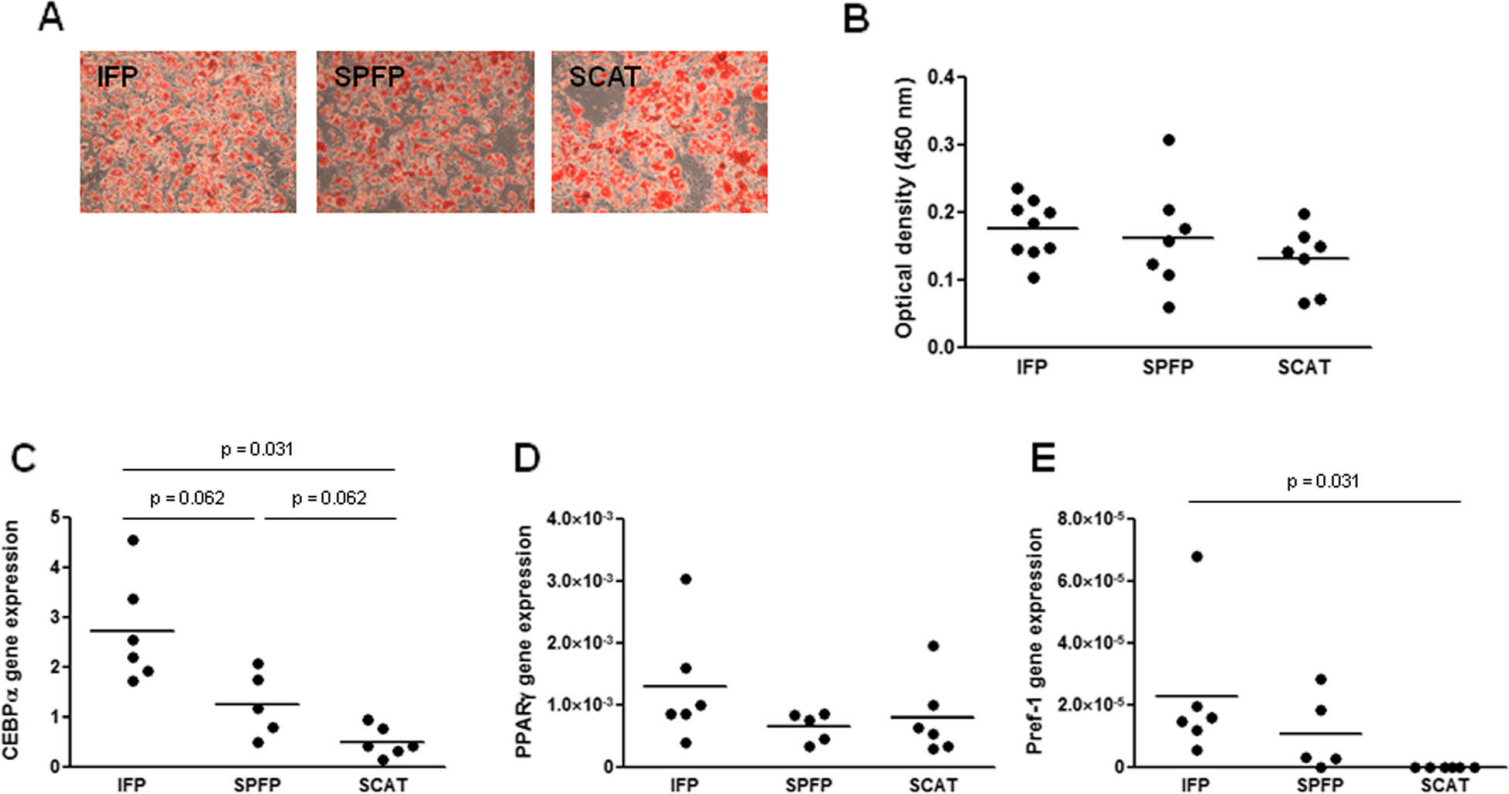
Adipocyte differentiation of dedifferentiated fat cells isolated from IAATs and SCATs. **a**, **b** DFATc from IFPs, SPFPs, and SCATs of OA patients (*n* = 7–9) were cultured for 14 days in adipogenic medium before the evaluation of adipocyte differentiation by oil red O staining (**a**) and staining quantification (**b**). **c**–**e** Relative mRNA expression of CEBPα (**c**), PPARγ (**d**), and Pref-1 (**e**) by DFATc isolated from IFPs and SPFPs compared to the corresponding SCAT-derived DFATc from OA patients (*n* = 5–6). The relative mRNA expression was normalized to that of 18S RNA. Bars indicate the mean expression levels

**Fig. 4:**
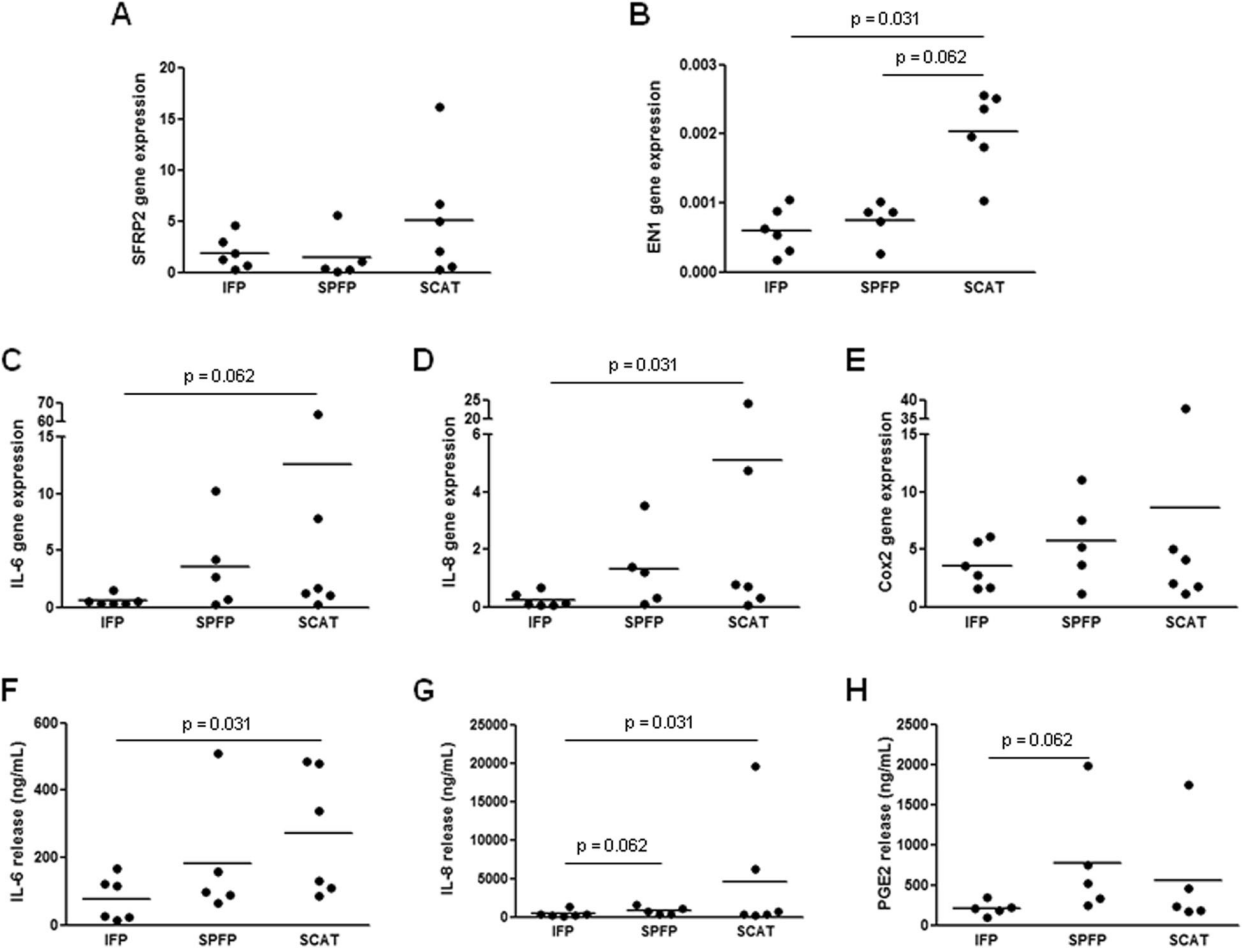
Molecular characterization of dedifferentiated fat cells isolated from IAAT and SCAT. **a**–**e** Relative mRNA expression of SFRP2 (**a**), EN-1 (**b**), IL-6 (**c**), IL-8 (**d**), and Cox2 (**e**) by IFP- and SPFP-derived dedifferentiated adipocytes compared to the corresponding SCAT-derived DFATc from OA patients (*n* = 5–6). The relative mRNA expression was normalized to that of 18S RNA. **f**–**h** The secretion of IL-6 (**f**), IL-8 (**g**), and PGE_2_ (**h**) by DFATc isolated from IFPs, SPFPs, and SCATs (*n* = 5–6). Bars indicate the mean expression levels

DFATc cultures from IFPs, SPFPs, and SCATs expressed CD90 and CD105 and were negative for CD45 and CD31 (Additional file 2: Figure S2). These cells were able to differentiate into adipocytes, and the tissue origin did not affect the differentiation (Fig. 3a, b). As we observed in preadipocytes, DFATc from IFPs and SPFPs expressed more C/EBPα than SCAT DFATc (5.4-fold, *p* = 0.031, and 2.5-fold, *p* = 0.062, for IFP and SPFP DFATc, respectively), while the mRNA expression of PPARγ was similar in DFATc from the different ATs (Fig. 3c, d). Pref-1 mRNA was expressed by the majority of cultures isolated from IFPs (5/5 patients) and SPFPs (4/5 patients), whereas no cultures from SCATs expressed Pref-1 (Fig. 3e). The mRNA expression level of EN-1 was lower in DFATc isolated from IAATs than from SCATs (*p* = 0.031 and *p* = 0.062 for IFP- and SPFP- derived DFATc, respectively), whereas SFRP2 expression was similar (Fig. 4a, b). The expression pattern of the inflammatory factors by IFP-, SPFP-, and SCAT-derived DFATc and preadipocytes was also similar (Fig. 4c–h). IFP DFATc showed the lowest basal expression and release of IL-6 and IL-8 (*p* = 0.062 and *p* = 0.031 for the mRNA expression of IL-6 and IL-8 and *p* = 0.031 and *p* = 0.031 for the release of IL-6 and IL-8 compared to cells from SCATs) (Fig. 4c, d, f, g). No significant difference in IL-6 and IL-8 release between IFP- and SPFP-derived DFATc was found (Fig. 4f, g). No difference in the basal mRNA expression and secretion of inflammatory factors by SPFP- and SCAT-derived DFATc was observed (Fig. 4c–h).

These results show that the molecular characteristics of DFATc and preadipocytes is similar, suggesting that the phenotypic differences between preadipocytes from IAATs and SCATs result from differences in the intrinsic properties of these cells rather than from heterogeneity in the mesenchymal cell population isolated from each AT.

### Stronger response to inflammatory stimulation in preadipocytes and dedifferentiated fat cells from IAATs

**Fig. 5:**
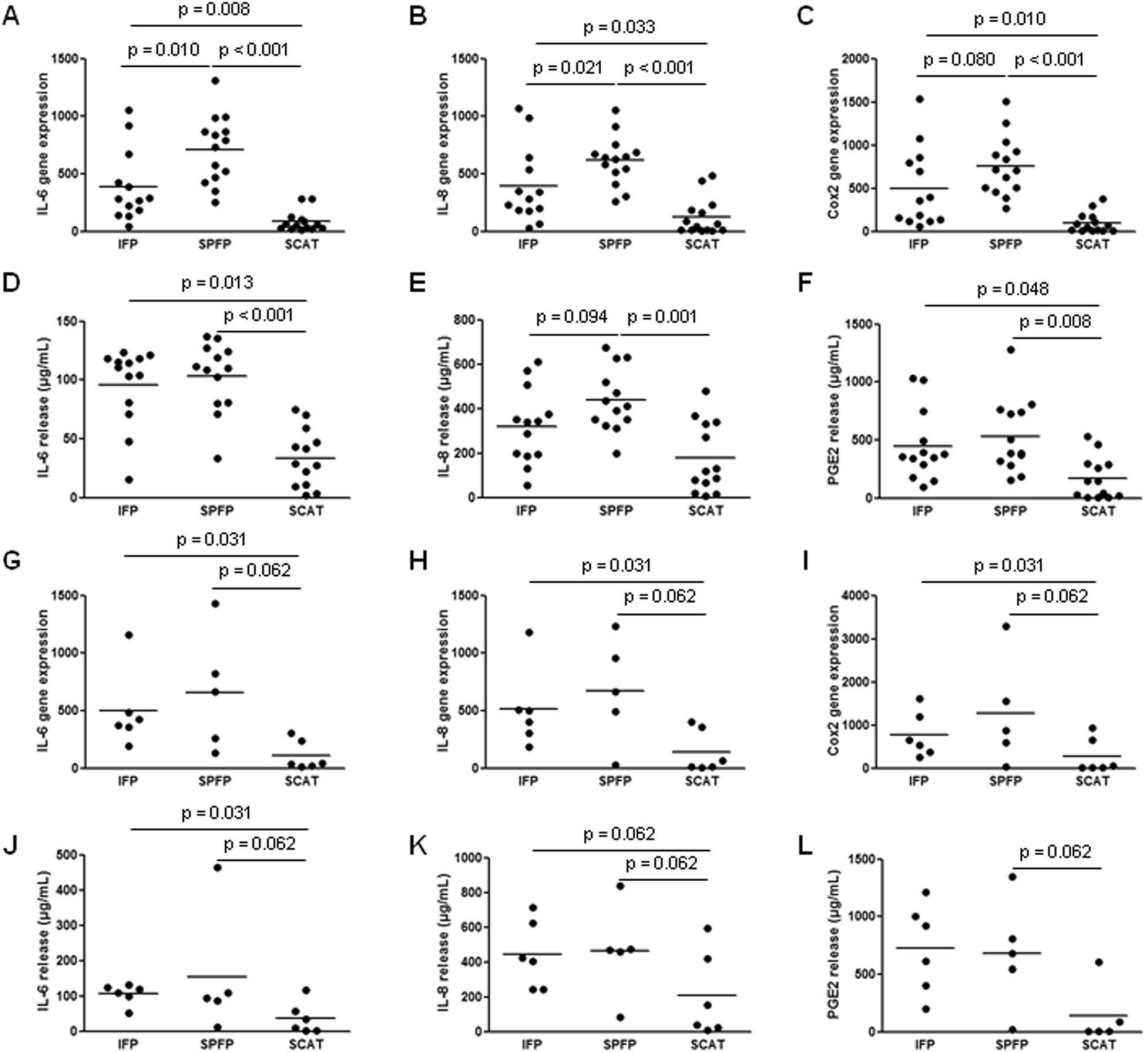
Response of preadipocytes and dedifferentiated fat cells to IL-1β stimulation. Preadipocytes (*n* = 13) (**a**–**f**) and DFATc (*n* = 6) (**g**–**l**) isolated from the IFPs and SCATs of OA patients were stimulated by IL-1β (1 ng/mL) before RT-qPCR analysis of the mRNA expression of IL-6 (**a**, **g**), IL-8 (**b**, **h**), and Cox2 (**c**, **i**). The relative mRNA expression was normalized to that of 18S RNA. The IL-6 (**d**, **j**), IL-8 (**e**, **k**), and PGE_2_ (**f**, **l**) that were released into cell conditioned medium were measured by ELISAs. Bars indicate the mean expression levels

As IAATs in OA are enriched in inflammatory cells and are adjacent to the synovium, where inflammation is mainly concentrated, the environment of AT resident is inflammatory. We first studied the response of preadipocytes and DFATc to IL-1β stimulation (Fig. 5). Regardless of the AT from which preadipocytes and DFATc were isolated, IL-1β stimulated the mRNA expression of IL-6, IL-8, and Cox2 and the release of IL-6, IL-8, and PGE_2_ (Fig. 5). However, the effect of IL-1β was significantly more potent on cells isolated from IAATs (Fig. 5a–l). IL-1β stimulated 4.6- (*p* = 0.008), 3.1- (*p* = 0.033), and 5.0-fold (*p* = 0.010) more mRNA expression of IL-6, IL-8, and Cox2, respectively, in IFP preadipocytes than in SCAT preadipocytes (Fig. 5a–c). Similarly, the expression of IL-6, IL-8, and Cox2 in response to IL-1β was increased by 8.4- (*p* < 0.001), 4.9- (*p* < 0.001), and 7.6-fold (*p* < 0.001) in SPFP preadipocytes compared to that in SCAT preadipocytes (Fig. 5a–c). The release of IL-6, IL-8, and PGE_2_ by IFP and SPFP preadipocytes was consequently higher than that by SCAT preadipocytes after IL-1β stimulation (Fig. 5d–f). In response to IL-1β stimulation, SPFP-derived preadipocytes expressed more IL-6 (*p* = 0.010) and IL-8 (*p* = 0.021) than IFP-derived preadipocytes (Fig. 5a, b). Similar results were obtained with DFATc (Fig. 5g–l).

**Fig. 6:**
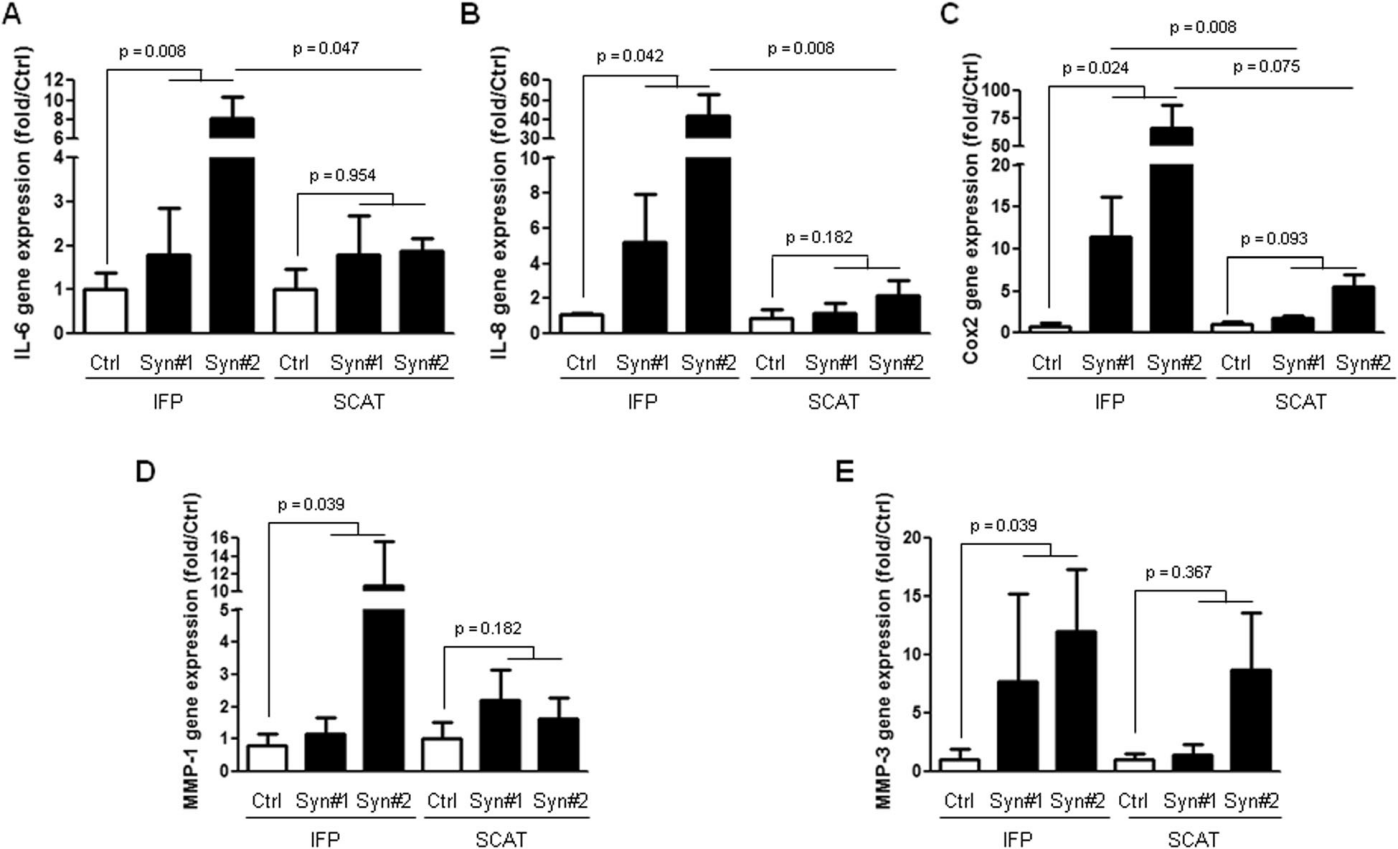
Response of IFP and SCAT preadipocytes to stimulation by conditioned media of the synovium from OA patients. Preadipocytes isolated from the IFPs and SCATs of OA patients (*n* = 4 to 5) were stimulated or not (Ctrl) with conditioned media from the inflamed part of synovium from two OA patients (Syn#1 and Syn#2) before the RT-qPCR analysis of the mRNA expression of IL-6 (**a**), IL-8 (**b**), Cox2 (**c**), MMP-1 (**d**), and MMP-3 (**e**). The relative mRNA expression was normalized to that of 18S RNA. Data are expressed as the relative expression of the genes of interest to control unstimulated cells. Error bars are SD. Statistical analysis compared the effect of stimulation by conditioned media from inflamed OA synovium to control unstimulated preadipocytes, using the non-parametric Friedman and Dunn’s multiple comparison tests. The effect of Syn#1 and Syn#2 conditioned media on IFP-derived preadipocytes relative to SCAT-derived preadipocytes was assessed using the non-parametric non-paired Mann-Whitney test

We next investigated the response of IFP and SCAT preadipocytes to stimulation by conditioned media of synovium from OA patients (Fig. 6). As synovial inflammation usually appears in patchy areas in OA, we used conditioned media from inflamed areas, known to release higher levels of inflammatory factors, to stimulate preadipocytes [19]. As previously observed in response to IL-1β stimulation, a stronger response was obtained when IFP rather than SCAT preadipocytes were stimulated. The stimulation by conditioned media from OA synovium increased the mRNA expression of IL-6 (*p* = 0.008), IL-8 (*p* = 0.042), Cox2 (*p* = 0.024), MMP-1 (*p* = 0.039), and MMP-3 (*p* = 0.039) by IFP preadipocytes. In contrast, no statistically significant increase in the mRNA expression by SCAT preadipocytes was observed. In addition, the stimulation by inflamed synovium conditioned media induced a higher increase in the mRNA expression of genes of interest in IFP-derived preadipocytes as compared to SCAT-derived preadipocytes and reached statistical significance for IL-6 (*p* = 0.047, for Syn#2), IL-8 (*p* = 0.008, for Syn#2), and Cox2 (*p* = 0.008, for Syn#1).

These results show that the phenotypic difference of OA preadipocytes from IAATs and SCATs includes altered responses to inflammatory stimulation, which is exacerbated in IAATs.

## Discussion

OA is a whole joint disease, meaning that functional interactions between joint tissues play a critical role. Thus, it is of crucial importance to understand the phenotype of the cells residing in each OA joint tissue to understand how these cells can impact joint homeostasis. In this context, much attention has been given to IAAT alterations in OA and on their consequences [1–5, 7, 8]. We and others have shown that IAATs from OA patients display specific features that differ from those of autologous SCATs [1, 2, 5]. Notably, IAATs induce an inflammatory and fibrotic program in OA fibroblast-like synoviocytes, supporting their involvement in OA [1, 2, 20]. In the present study, we studied whether IAAT preadipocytes of OA patients display unique features, which would support their involvement in OA. We isolated preadipocytes from the SVF of IFPs and SPFPs and compared their characteristics to those of preadipocytes isolated from autologous SCATs. Cultured cells from either IFPs, SPFPs, or SCATs were analyzed by flow cytometry to confirm their mesenchymal origin and to confirm the absence of endothelial and leukocyte marker expression. Because the differences observed between mesenchymal cells isolated from the SVF of IAATs and SCATs may be due to a difference in the amount of preadipocytes within the pool of cells rather than due to a true phenotypic difference between the preadipocytes of each tissue, we performed the same analyses on DFATc. These DFATc are a more homogeneous cell population and show similar properties to MSCs [18, 21]. We found similar results for preadipocytes and DFATc, suggesting that the differences we highlighted between IAAT and SCAT preadipocytes resulted from intrinsic phenotypic differences in the preadipocytes of these tissues. Nevertheless, although DFATc are considered to come from isolated floating mature adipocytes [18], the presence of other cells aggregated with adipocytes cannot be completely excluded [22].

Our study is the first to highlight a difference in the potential of IAAT and SCAT precursors to differentiate into mature adipocytes. Indeed, we show that preadipocytes from human IFPs and SPFPs have a greater ability to differentiate into adipocytes than the precursors of autologous SCATs. Previous animal and human studies have already shown specific differentiation properties of adipose stem cells (ASCs) that depend on their origin [13, 23–26]. Thus, ASCs from SCATs have a greater adipogenic potential than ASCs from visceral ATs, while no difference was observed between human ASCs from OA IFPs and SCATs [13, 27]. The discrepancies between our result and those previously published may come from differences in the protocols used for adipocyte differentiation. Nevertheless, the large number of patients included in our study, the use of two different IAATs (IFP and SPFP), and the analysis of two different cellular models (preadipocytes and DFAT cells) make our results fairly robust. Furthermore, although Lopa et al. did not observe any difference in adipocyte differentiation between IFP- and SCAT-derived precursors, they do revealed differences in their ability of chondrocyte and osteoblast differentiation, showing that IFP- and SCAT-derived precursors display distinct phenotype [13].

Grandl et al. proposed that the composition of extracellular matrix surrounding the ASCs rather than the ASCs themselves explains this difference and pointed out a role for collagen fibers [24]. In the present study, we did not assess the impact of the cellular microenvironment on adipocyte precursor properties. However, we previously reported an increased amount of extracellular collagen deposits in IAATs, which could influence the phenotype of IAAT resident cells [2].

Unlike Grandl et al., we show that preadipocytes have specific molecular characteristics that depend on their tissue origin [24]. Consistent with their increased adipocyte differentiation ability, IAAT preadipocytes showed stronger expression of C/EBP-α and PPARγ mRNA than SCAT-derived preadipocytes. IAAT preadipocytes also expressed higher levels of pref-1, a marker of adipogenic commitment [28]. In addition to genes whose function is linked to adipocyte differentiation, preadipocytes isolated from IAATs and SCATs also have a distinct expression pattern for both EN1 and SFRP2, two development-related genes. Indeed, the expression of EN1 and SFRP2 was lower in IAAT preadipocytes than in SCAT preadipocytes. In a previous study, we reported that EN1 expression was lower in the whole knee and hip IAATs (IFPs, SPFPs, and acetabular fat pads) than in autologous SCATs from OA patients, whereas the expression of SFRP2 was slightly but not significantly increased in knee and hip IAATs [2]. Similar differences were observed between visceral ATs and SCATs [29]. The characteristics also differ between IAATs and SCATs [2, 5]. Together, these results indicate that IAATs and SCATs display different features when whole tissue or isolated preadipocytes and adipocytes are examined [2, 5], suggesting that the unique phenotype of preadipocytes is maintained by mature adipocytes, the main cell type of ATs. Further studies should investigate whether IAAT adipocytes retain the specific phenotype of their precursors.

Since IAAT and SCAT preadipocytes are phenotypically distinct and IAATs produce more inflammatory factors than SCATs [1, 2, 5, 7], we investigated whether this feature of IAATs is also present in the corresponding preadipocytes. When cultured under basal conditions, IAAT preadipocytes express either similar or lower amounts of inflammatory factors than SCAT preadipocytes. In contrast, IAAT preadipocytes have an exacerbated response to inflammatory stimulation by IL-1β or by the conditioned media of OA synovium compared to SCAT preadipocytes. This stronger responsiveness to inflammatory stimulation could partially explain the higher production of inflammatory factors by IAATs than by SCATs [1, 2, 5, 7]. Indeed, inflammatory factors released by leukocytes, which are more numerous in IAATs than SCATs, could stimulate preadipocytes and induce an inflammatory response, which would then be stronger in IAATs than in SCATs.

OA is characterized by a chronic inflammatory state that particularly affects the synovium [30, 31]. Indeed, OA synovial membrane is the site of heterogeneous inflammatory infiltrate, with some areas macroscopically more inflammatory than others. We previously showed that inflammatory part of OA synovium secreted higher amount of most inflammatory factors analyzed than the non-inflammatory part [19]. Interestingly, among all the cytokines that we found overproduced by inflammatory OA synovium, IL-1β was the one with the strongest increase measured in the inflamed part of OA synovium, as compared to the non-inflamed one.

IAATs from early to late OA are also characterized by a chronic inflammatory state [2, 32, 33]. The closed contact between IAATs and synovium allowed paracrine functional interactions between these tissues. We and others have already shown that IAAT conditioned media were able to induce a proinflammatory and profibrotic response in autologous synoviocytes [1, 2, 20]. Here, we observed that OA synovium-conditioned media also promoted a proinflammatory status in IAAT preadipocytes, close to that induced by IL-1β, the main cytokine induced by the inflammatory state of OA synovium [19]. These results highlight a reciprocal action between the synovium and IAATs and strongly support viewing the synovium and IAATs as a functional unit within the joint.

OA induces changes in IAATs at the tissue and molecular levels. IAATs from patients with OA and anterior cruciate ligament rupture show distinct phenotypes [8]. However, to the best of our knowledge, no study has focused on the impact of inflammatory stimulation on the phenotype of IAAT preadipocytes based on the OA status. Recently, the expression of genes involved in chondrogenesis by ASCs isolated from OA and non-OA IFPs in response to OA synovial fluid was compared [34]. Modifications in gene expression were observed in only non-OA ASCs, suggesting that OA has an impact on the phenotype of adipocyte precursors. Further studies should be performed on non-OA IAAT preadipocytes to confirm whether their specific phenotype is acquired or intrinsic.

In conclusion, our results show that IAAT preadipocytes shared unique phenotypes that distinguished these cells from SCAT preadipocytes. The ability of IAAT preadipocytes to differentiate into adipocytes is higher than that of SCAT preadipocytes, and IAAT preadipocytes have a molecular expression pattern that is different from that of SCAT preadipocytes. Importantly, IAAT preadipocytes display an exacerbated response to inflammatory stimulation compared to that of SCAT preadipocytes, which may have large consequences, especially on the adjacent synovium. These data are also of interest to the growing field of OA treatment by MSCs, which can be isolated from IAATs. The intra-articular injection of cells with an exacerbated response to inflammatory stimulation into an inflamed environment could be deleterious rather than protective. This hypothesis should be tested before the clinical development of these treatment methods.

## Supplementary information

**Additional file 1: Figure S1.** Protocol of isolation and culture of preadipocytes and dedifferentiated fat cells from adipose tissues. After AT digestion and filtration, the centrifugation step separates the stromal vascular fraction (SVF) and mature adipocytes, which sediments at the bottom and float at the top of the tube, respectively. The suspension of mature adipocytes was then placed in a culture flask (25 cm^2^), which was completely filled to allow the floating adipocytes to contact the adhesive surface of the flask, as described by Matsumoto et al. [9]. Some adipocytes attached to the “ceiling” of the flask, initiating a dedifferentiation process and becoming dedifferentiated fat cells (DFATc). After 7 days, the medium was changed, and the flasks were inverted to allow DFATc proliferation to continue. After resuspension and lysis of red blood cells, the SVF containing preadipocytes was seeded in a culture flask and cultured in a medium allowing proliferation of preadipocytes.

**Additional file 2: Figure S2.** Flow cytometry analysis of preadipocytes and DFATc from IAATs and SCAT of OA patients. Cell surface expression of CD31 (a-f), CD45 (g-l), CD90 (m-r) and CD105 (s-x) by preadipocytes (a-c, g-i, m-o and s-u), and DFATc (d-f, j-l, p-r and v-x) isolated from IFP (a, d, g, j, m, p, s and v), SPFP (b, e, h, k, n, q, t and w) and SCAT (c, f, i, l, o, r, u and x). Red curves represent the expression of markers by IAAT and SCAT-derived cells, whereas black curves indicate the negative control. Preadipocytes and DFATc isolated from IFP, SPFP and SCAT showed an expression of CD90 and CD105, whereas they did not express CD31 and CD45.

**Additional file 3: Table S1.** Characteristics of knee OA patients. **Table S2.** Sequence of primers used for RT-PCR studies.

## Data Availability

The datasets used and/or analyzed during the current study are available from the corresponding author on reasonable request.

## Abbreviations

ASCs: Adipose stem cells
ATs: Adipose tissues
DFATc: Dedifferentiated fat cells
DMEM: Dulbecco’s modified Eagle’s medium
EDTA: Ethylene diamine tetraacetic acid
FCS: Fetal calf serum
IAATs: Intra-articular adipose tissues
IFP: Infrapatellar fat pad
IL: Interleukin
MSCs: Mesenchymal stem cells
OA: Osteoarthritis
PFA: Paraformaldehyde
PGE_2_: Prostaglandin E2
SCATs: Subcutaneous adipose tissues
SPFP: Suprapatellar fat pad
SVF: Stromal vascular fraction
