# Supplementary figures and images for: Contribution of adipocyte precursors in the phenotypic specificity of intra-articular adipose tissues in knee osteoarthritis patients
Source: Arthritis Res Ther. 2019 Nov 27;21:252. doi: 10.1186/s13075-019-2058-9 (PMC6882235; doi:10.1186/s13075-019-2058-9)

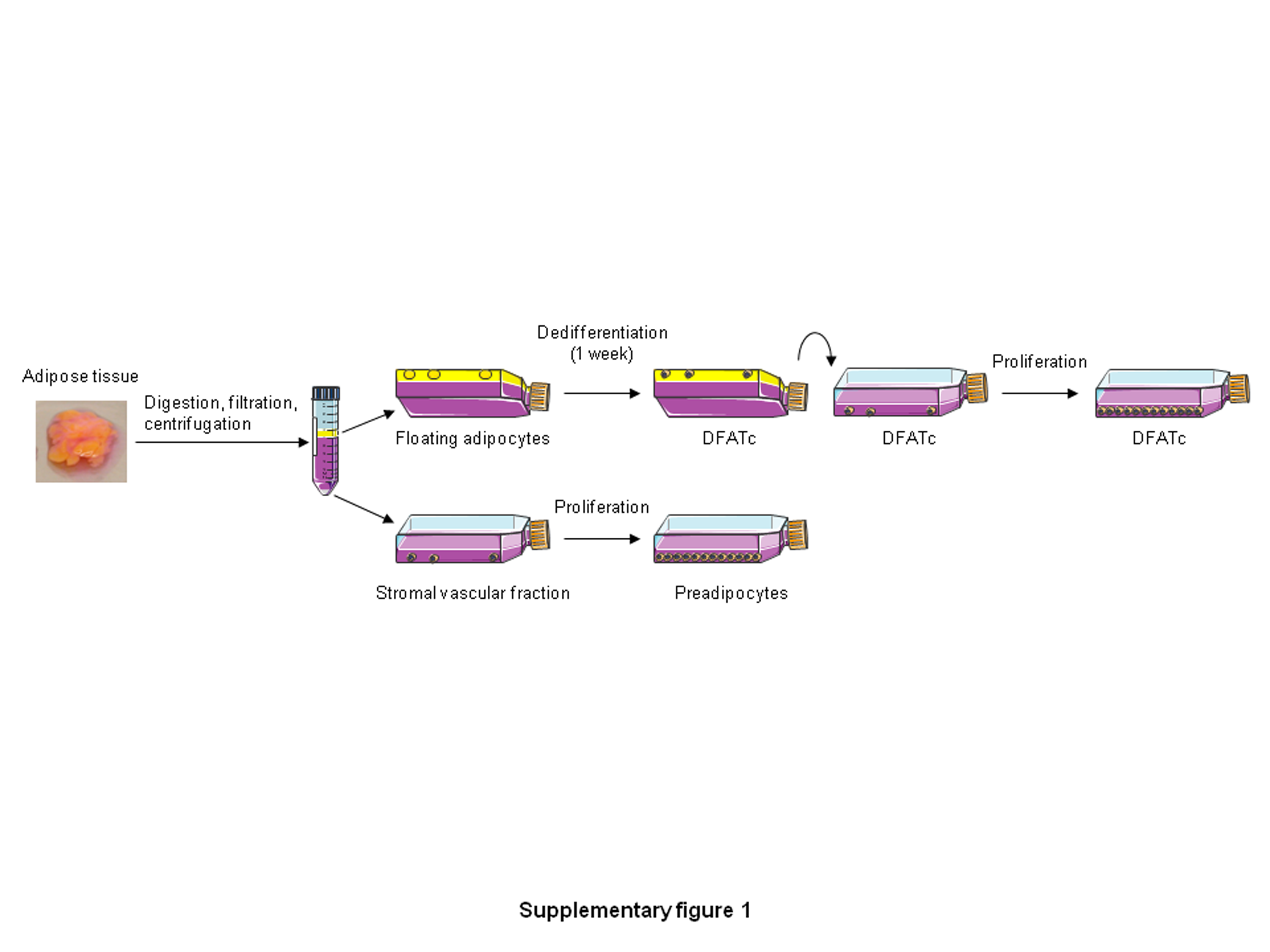

Supplement: Supplementary file 1 — Additional file 1: Figure S1. Protocol of isolation and culture of preadipocytes and dedifferentiated fat cells from adipose tissues. After AT digestion and filtration, the centrifugation step separates the stromal vascular fraction (SVF) and mature adipocytes, which sediments at the bottom and float at the top of the tube, respectively. The suspension of mature adipocytes was then placed in a culture flask (25 cm2), which was completely filled to allow the floating adipocytes to contact the adhesive surface of the flask, as described by Matsumoto et al. [9]. Some adipocytes attached to the “ceiling” of the flask, initiating a dedifferentiation process and becoming dedifferentiated fat cells (DFATc). After 7 days, the medium was changed, and the flasks were inverted to allow DFATc proliferation to continue. After resuspension and lysis of red blood cells, the SVF containing preadipocytes was seeded in a culture flask and cultured in a medium allowing proliferation of preadipocytes. [file 13075_2019_2058_MOESM1_ESM.tif]

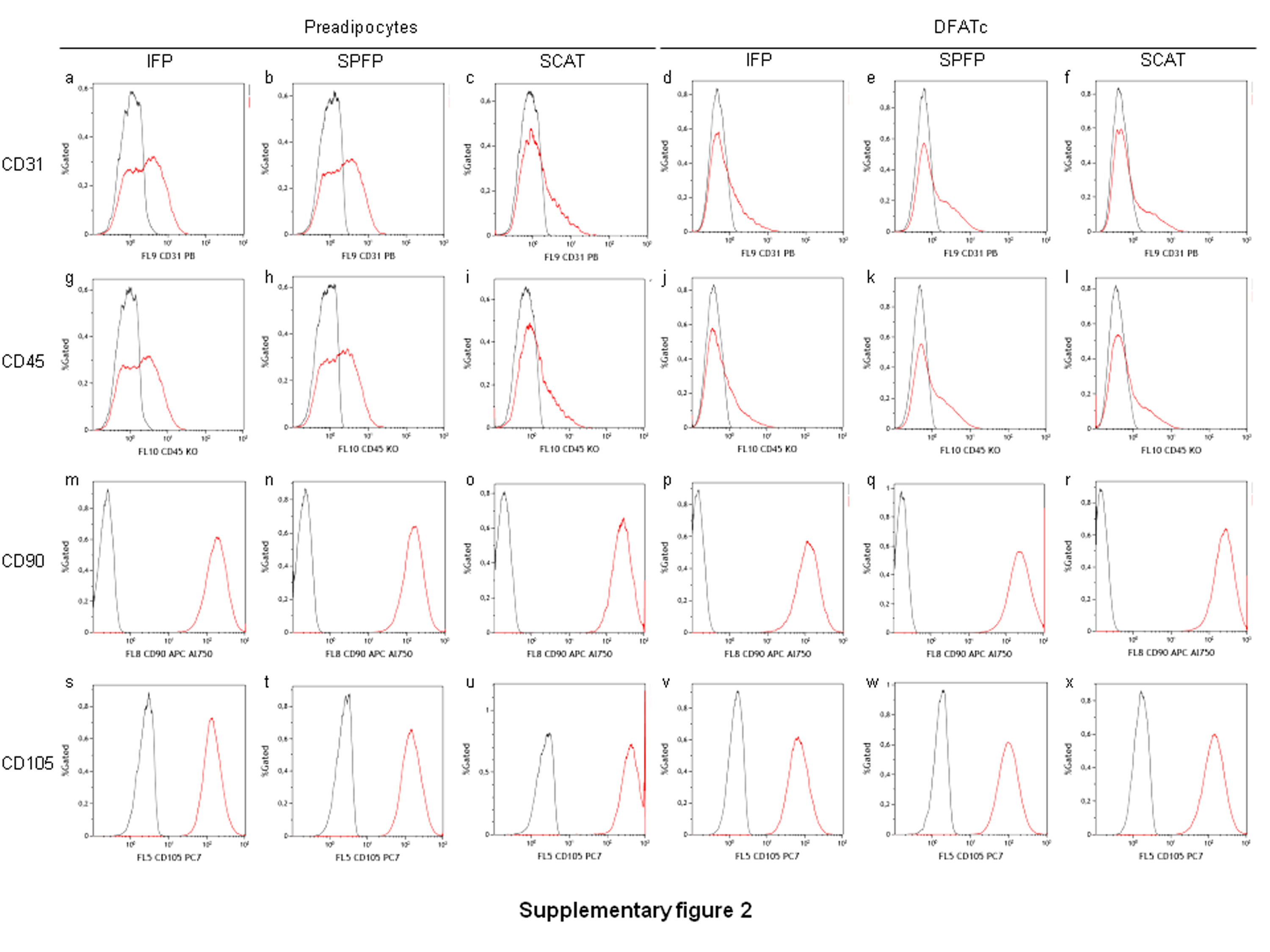

Supplement: Supplementary file 2 — Additional file 2: Figure S2. Flow cytometry analysis of preadipocytes and DFATc from IAATs and SCAT of OA patients. Cell surface expression of CD31 (a-f), CD45 (g-l), CD90 (m-r) and CD105 (s-x) by preadipocytes (a-c, g-i, m-o and s-u), and DFATc (d-f, j-l, p-r and v-x) isolated from IFP (a, d, g, j, m, p, s and v), SPFP (b, e, h, k, n, q, t and w) and SCAT (c, f, i, l, o, r, u and x). Red curves represent the expression of markers by IAAT and SCAT-derived cells, whereas black curves indicate the negative control. Preadipocytes and DFATc isolated from IFP, SPFP and SCAT showed an expression of CD90 and CD105, whereas they did not express CD31 and CD45. [file 13075_2019_2058_MOESM2_ESM.tif]
