# Supplementary material for: Contribution of adipocyte precursors in the phenotypic specificity of intra-articular adipose tissues in knee osteoarthritis patients
Source: Arthritis Res Ther. 2019 Nov 27;21:252. doi: 10.1186/s13075-019-2058-9 (PMC6882235; doi:10.1186/s13075-019-2058-9)
Supplement: Supplementary file 3 — Additional file 3: Table S1. Characteristics of knee OA patients. Table S2. Sequence of primers used for RT-PCR studies. [file 13075_2019_2058_MOESM3_ESM.docx]

**Table S1. Characteristics of knee OA patients.**

|  | All patients | Patients for preadipocyte experiments | Patients for DFAT cell experiments |
| --- | --- | --- | --- |
| Number of patients | 20 | 17 | 9 |
| Age, years | 72.1 ± 9.9 | 71.6 ± 10.0 | 73.6 ± 12.8 |
| Gender (Female), n (%) | 16 (80.0) | 14 (82.4) | 6 (66.7) |
| Weight, kg | 75.8 ± 15.8 | 76.6 ± 15.1 | 76.4 ± 19.0 |
| Height, cm | 161.7 ± 7.2 | 161.6 ± 7.2 | 164.4 ± 8.9 |
| Body mass index, kg/m^2^ | 28.4 ± 5.0 | 28.7 ± 5.1 | 26.9 ± 4.3 |
| Hypertension, n (%) | 15 (75.0) | 12 (70.6) | 7 (77.8) |
| Diabetes, n (%) | 2 (10.0) | 2 (11.8) | 0 (0.0) |
| Dyslipidemia, n (%) | 9 (45.0) | 9 (52.9) | 2 (22.3) |
| Metabolic syndrome, n (%) | 8 (40.0) | 8 (47.1) | 2 (22.3) |

**Table S2. Sequence of primers used for RT-PCR studies.**

| **Gene** | **Forward primer (5’-3’)** | **Reverse primer (5’-3’)** |
| --- | --- | --- |
| **18s** | GCAATTATTCCCCATGAACG | GGGACTTAATCAACGCAAGC |
| **IL-6** | CAATGAGGAGACTTGCCTGG | GCACAGCTCTGGCTTGTTCC |
| **IL-8** | TGACTTCCAAGCTGGCCGTG | CGCAGTGTGGTCCACTCTCA |
| **Cox2** | GTTCCACCCGCAGTACAGAA | AGGGCTTCAGCATAAAGCGT |
| **MCP1** | AGTCTCTGCCGCCCTTCT | GTGACTGGGGCATTGATTG |
| **MMP1** | AGTGGCCCAGTGGTTGAAAA | CCACATCAGGCACTCCACAT |
| **MMP3** | CCCAAGAGGCATCCAC | GGGTCAAACTCGAACTGT |
| **SFRP2** | GCCTCGATGACCTAGACGAG | GGTCGCACTCAAGCATGTC |
| **EN1** | TCGGACAGGTGCTATCGAA | AAGGAGTTCGCAGTTTCGTC |
| **PPARγ** | GACAGGAAAGACAACAGACAAATC | GGGGTGATGTGTTTGAACTTG |
| **C/EBPα** | ACTGGGACCCTCAGCCTTG | TGGACTGATCGTGCTTCGTG |
| **Pref-1** | GAAAGGACTGCCAGAAAAAGG | GCAGAAATTGCCTGAGAAGC |
